# Contributions of the Epidermal Growth Factor Receptor to Acquisition of Platinum Resistance in Ovarian Cancer Cells

**DOI:** 10.1371/journal.pone.0136893

**Published:** 2015-09-09

**Authors:** Michaela L. Granados, Laurie G. Hudson, Sabrina L. Samudio-Ruiz

**Affiliations:** Department of Pharmaceutical Sciences, College of Pharmacy, University of New Mexico, Albuquerque, NM, United States of America; University of Louisville, UNITED STATES

## Abstract

Acquisition of platinum resistance following first line platinum/taxane therapy is commonly observed in ovarian cancer patients and prevents clinical effectiveness. There are few options to prevent platinum resistance; however, demethylating agents have been shown to resensitize patients to platinum therapy thereby demonstrating that DNA methylation is a critical contributor to the development of platinum resistance. We previously reported the Epidermal Growth Factor Receptor (EGFR) is a novel regulator of DNA methyltransferase (DNMT) activity and DNA methylation. Others have shown that EGFR activation is linked to cisplatin treatment and platinum resistance. We hypothesized that cisplatin induced activation of the EGFR mediates changes in DNA methylation associated with the development of platinum resistance. To investigate this, we evaluated EGFR signaling and DNMT activity after acute cisplatin exposure. We also developed an *in vitro* model of platinum resistance to examine the effects of EGFR inhibition on acquisition of cisplatin resistance. Acute cisplatin treatment activates the EGFR and downstream signaling pathways, and induces an EGFR mediated increase in DNMT activity. Cisplatin resistant cells also showed increased DNMT activity and global methylation. EGFR inhibition during repeated cisplatin treatments generated cells that were more sensitive to cisplatin and did not develop increases in DNA methylation or DNMT activity compared to controls. These findings suggest that activation of EGFR during platinum treatment contributes to the development of platinum resistance. Furthermore, EGFR inhibition may be an effective strategy at attenuating the development of platinum resistance thereby enhancing the effectiveness of chemotherapeutic treatment in ovarian cancer.

## Introduction

Ovarian cancer is the leading cause of death arising from gynecological malignancies [1]. Advanced disease, late stage diagnosis, peritoneal metastasis and frequent development of chemoresistance impede improvements in the overall survival rate which remains low at roughly 44% [1]. First line treatment for ovarian cancer includes surgical debulking and platinum (cisplatin or carboplatin)–taxane (paclitaxel) chemotherapy [2]. As many as 70–80% of ovarian cancer patients will develop platinum resistance after first line therapy and most of these patients eventually succumb to chemoresistant disease [3–5]. Thus, platinum resistance continues to be a significant clinical challenge. To date, there are limited interventions available to prevent or reverse platinum resistance; however, there have been some advances in the use of demethylating agents in the resensitization of patients to platinum based therapy [6–10]. Specifically, Matei and colleagues showed that platinum resistant patients treated with a low dose demethylating agent induced demethylation of genes within tumor cells and positively correlated with progression free survival [7]. This highlights DNA methylation as a critical contributor to the acquisition of drug resistance in ovarian cancer. However, mechanisms regulating DNA methylation and the acquisition of platinum resistance following cisplatin treatment have not been fully elucidated. We previously reported that the Epidermal Growth Factor Receptor (EGFR) regulates of DNA methyltransferases (DNMT) and DNA methylation [11]. Therefore, the EGFR may contribute to the development of platinum resistance.

The EGFR is a receptor tyrosine kinase that is overexpressed in 30–98% of epithelial ovarian cancer [4,5] and overexpression of EGFR (and its ligands) in ovarian cancer patients correlate with poor prognosis [12]. Activation of the EGFR in ovarian tumors is associated with increased malignancy and poor patient outcome [13,14]. Furthermore, activation of EGFR has been shown in ~30% of ovarian tumors [15]. The EGFR is responsible for activation of multiple intracellular signaling pathways including Ras/Raf/MAPK, Jak/Stat and AKT/PI3K and regulates many cellular processes such as cell survival, proliferation and migration (see [14] for review). In addition, EGFR activation occurs in response to cisplatin [16–19] and hyperactivation of the receptor, and its downstream signaling pathways, is implicated in platinum resistance [20,21]. We previously showed that activation of the EGFR in ovarian cancer cells increases DNMT activity and over long term EGFR activation can lead to increased DNA methylation [11] as well as decreased sensitivity to cisplatin [22].

Platinum or cisplatin resistance is correlated with increased DNA methylation and subsequent silencing of genes involved in appropriate drug response [23–28]. Gene expression analysis of platinum sensitive versus platinum resistant patient samples showed that the differentially regulated genes are more likely to be underexpressed in resistant compared to sensitive tumors [29]. Taken together, we hypothesized that the cisplatin induced activation of the EGFR contributes to the development of platinum resistance in ovarian cancer cells through regulation of DNMT activity and DNA methylation. Furthermore, we suggest that small molecule inhibitors to the EGFR may be useful at preventing or diminishing the acquisition of cisplatin resistance. To test our hypothesis, we evaluated activation of the EGFR, downstream signaling pathways and DNA methyltransferase activity in ovarian cancer cells in response to physiologically relevant doses of cisplatin. We also investigated the effects of the small molecule inhibitor Erlotinib in an *in vitro* model of platinum resistance. We found that inhibition of the EGFR attenuates cisplatin induced increases in DNMT activity, prevents increased DNA methylation and also diminishes platinum resistance in ovarian cancer cells. This work highlights a potential mechanism and a tractable target to reduce the development of platinum resistance based on epigenetic mechanisms.

## Materials and Methods

### Cell culture and drug treatment

The ovarian carcinoma cell line OVCA 433 was provided by Dr. Robert Bast Jr., M.D. Anderson Cancer Center, Houston TX [30] and grown in Minimum Essential Medium (MEME) (Gibco, Life Technologies, Grand Island, NY) supplemented with 10% (v/v) fetal bovine serum (FBS) (Atlanta Biologicals, Lawrenceville, GA), 1 mM sodium pyruvate (Sigma, St. Louis, MO), 0.5 units/mL penicillin-0.5 μg/mL streptomycin (Gibco, Life Technologies), later referred to as MEME growth media. All Cells were maintained at 37°C under 5% CO2/95% air. Acute 10 μM cisplatin (Sigma-Aldrich) treatments were done in MEME growth media for 0–6 hours. EGFR inhibition was carried out by way of preincubation of the cells with 2 μM AG1478 (LC Laboratories, Woburn, MA) for 24 hours prior to cisplatin treatment. 10 nM EGF (Biomedical Technologies, Stoughton, MA) treatments for 15 minutes were performed as a positive control for EGFR activation.

### Cisplatin resistance paradigm

As originally demonstrated in [20], resistance to cisplatin can be increased in ovarian cancer cells by repeated sequential treatments with cisplatin followed by drug free recovery times. Our cisplatin resistance paradigm was modeled from [20]. Briefly, OVCA 433 cells were treated with cisplatin for 48 hours then allowed to recover for at least 48 hours after drug treatment. Cells completed three cycles of each concentration of cisplatin followed by drug free recovery times before being exposed to the next higher dose. The doses of cisplatin used were 3, 6 and 9 μM. Cells completing this paradigm were termed Cisplatin resistant (CPR) cells. Passage control cells (not undergoing cisplatin treatments) were carried out in parallel. EGFR was inhibited by treating cells with 1 μM Erlotinib (Cell Signaling, Danvers, MA) for at least 1 hour prior to drug treatments with cisplatin. Erlotinib was maintained in the culture media for 48 hours with cisplatin treatments and then cells were allowed to recover from all drug treatment in MEME growth media during drug free intervals.

### Immunoblotting

Cells were washed with PBS and harvested in cell lysis buffer containing 5 mM EDTA, 2 mM EGTA, 1 mM Na_3_VO_4_, leupeptin 10 mg/ml, pepstatin A 10 mg/ml, 1 mM PMSF in PBS and 1% SDS. Total protein concentrations were determined using the BCA protein assay kit (Thermo Fisher Scientific, Rockford, IL). Equal amounts of total cell lysates (30 μg) were electrophoresed through 10% SDS-polyacrylamide, transferred to 0.45 μm nitrocellulose (Thermo Fisher Scientific) and blocked with 3% BSA. Blots were probed with rabbit polyclonal anti-phospho-EGFR (Tyr1068) (Cell Signaling) at 1:1000, rabbit polyclonal anti-total EGFR (Santa Cruz, Dallas, TX) at 1:500, rabbit monoclonal anti-phospho-JAK2 (Abcam, Cambridge, MA) at 1:1000, rabbit monoclonal anti-total JAK2 (Cell Signaling) at 1:1000, rabbit monoclonal anti-phospho AKT (Cell Signaling) at 1:1000, mouse monoclonal anti- total AKT (BD Transduction Laboratories, Franklin Lakes, NJ) at 1:500, mouse monoclonal anti-phospho-STAT3 (ser727) (Cell Signaling) at 1:1000, rabbit monoclonal anti-total STAT3 (Cell Signaling) 1:1000, rabbit polyclonal anti-phospho-ERK1/2 (Cell Signaling) at 1:2000, rabbit polyclonal anti-total ERK1/2 (Cell Signaling) at 1:2000, rabbit polyclonal anti-DNMT1 (New England Bio Labs, Ipswich, MA) at 1:750, rabbit polyclonal anti-DNMT3A (Cell Signaling) at 1:1000, rabbit polyclonal anti-DNMT3B (Abcam) at 1:1000, rabbit polyclonal anti-DNMT3L (Abcam) at 1:1000, rabbit polyclonal pDNMT1 (Ser714) (Millipore, Billerica, MA) at 1:250 and a mouse monoclonal anti-GAPDH antibody (Millipore) at 1:1000, which was used as a loading control. Blots were then incubated in the appropriate secondary antibody (Promega, Madison, WI) and the immunoreactive proteins were detected using SuperSignal West Pico or Femto Chemiluminescence (Thermo Fisher Scientific). Imaging of the blots and densitometry was accomplished using the Kodak Image Station 440 and related software (NEN Life Science Products, Boston, MA).

### Monolayer and Multicellular Aggregate (MCA) cell culture

Cells were plated at 2000 cells per well in 96 well flat bottom cell culture plates (monolayer culture) and 96 well Lipidure U bottom plates (NOF America Corporation, White Plains, NY) (MCA culture). Cells grew overnight at 37°C under 5% CO2/95% air. Images were taken using Olympus IX70 equipped with DP72 digital camera and imaging software.

### Cell viability

Cells grown in monolayer culture and as MCAs were treated with increasing doses of cisplatin [0–300 μM] for 48 hours. Cell viability was measured using PrestoBlue (Life Technologies) according to manufacturer’s protocol. Briefly, 10 μl of PrestoBlue reagent was added per 100 μl of media and incubated for 1 hour (monolayer) or 24 hours (MCAs). After the respective incubation times, top-read fluorescence (RFUs; excitation 555 nm & emission 585 nm) was measured using SpectraMax M2 plate reader and SoftMax Pro v5.4 software (Molecular Devices, Sunnyvale, CA). Calculations of IC_50_ values for cell viability assays were determined using GraphPad Prism Software 4.0 (San Diego, CA).

### DNMT activity assay

Nuclear extracts were isolated using the EpiQuik Nuclear Extraction Kit (Epigentek, Farmingdale, NY) according to the manufacturer’s protocol. Protein concentrations for nuclear extracts were determined using the BCA protein assay kit (Thermo Fisher Scientific). Total DNMT activity was determined using 20 μg total protein and the EpiQuik DNA Methyltransferase (DNMT) Activity/Inhibition Assay Kit (Epigentek) as recommended by the manufacturer. Each plate was read using a microplate reader at 450 nm. The amount of methylated substrate DNA detected by the kit is proportional to the DNMT enzymatic activity in our samples. DNMT activity is calculated by the following equation:

Activity(OD/h/mg)=sample OD-blank OD/(μg protein x initial incubation time in hours)*1000

Sample values were normalized to values obtained for control, untreated OVCA 433 cells within the same experiment and expressed relative to one.

### Global DNA methylation quantification

DNA was isolated from cells using the DNeasy Blood and Tissue Kit according to manufacturer’s protocol (Qiagen, Valencia, CA). Global DNA methylation was evaluated using 250 ng of DNA and the MethylFlash Methylated DNA Quantification Kit (Epigentek) according to the manufacturer’s protocol. The amount of 5-methylcytosine within each sample is determined by a colorimetric assay which is detected by microplate reader at 450 nm. Quantification of DNA methylation is calculated by the following equation:

Methylation = sample OD - blank OD/ (slope of standards x 2)

Sample values were normalized to values obtained for control, untreated OVCA 433 cells within the same experiment and expressed as a percentage increase from 100% (control).

### Graphing and statistical analysis

All data was evaluated in duplicate against untreated passage control cells and collected from at least 4 independent experiments, unless otherwise indicated. Data were graphed and analyzed using GraphPad Prism Software 4.0 (San Diego, CA) using one-way ANOVA or two-way ANOVA and Tukey’s, Dunnet’s post hoc analysis or Bonferroni’s correction where appropriate. Standard unpaired t-test also used when appropriate.

## Results

### Acute cisplatin treatment initiates EGFR signaling

**Fig 1:**
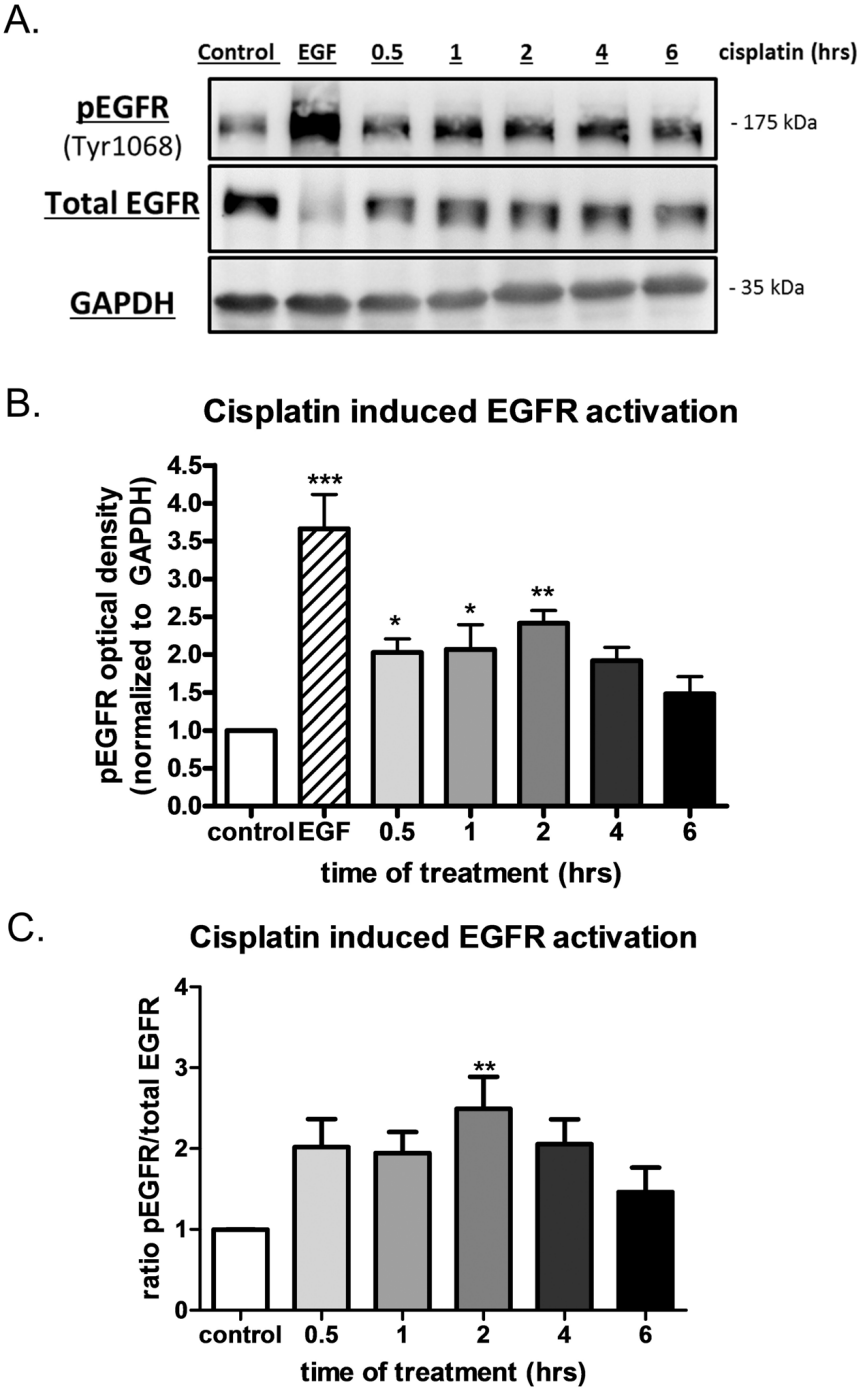
Acute cisplatin activates the Epidermal Growth Factor Receptor (EGFR). A) Representative western blots of activated or phosphorylated EGFR (pEGFR, tyr1068) in OVCA 433 cells given 10 μM cisplatin for 0.5, 1, 2, 4 and 6 hours compared to untreated control cells. 10 nM EGF given to cells for 15 minutes as a positive control showing activation of the receptor. Total EGFR blot and GAPDH representative blots also shown. B) Graph of EGFR activation following 10 μM cisplatin treatment for 0.5, 1, 2, 4 and 6 hours, n = 6. EGF data shown as a positive control. C) Graph showing ratio of pEGFR to total EGFR (pEGFR/total EGFR ratio) following 10 μM cisplatin treatment for 0.5, 1, 2, 4 and 6 hours, n = 6. One-way ANOVA of data revealed a significant effect of cisplatin treatment and significant differences from control as determined by Dunnet’s post hoc analysis indicated by *p<0.05, **p<0.01, ***p<0.001.

**Fig 2:**
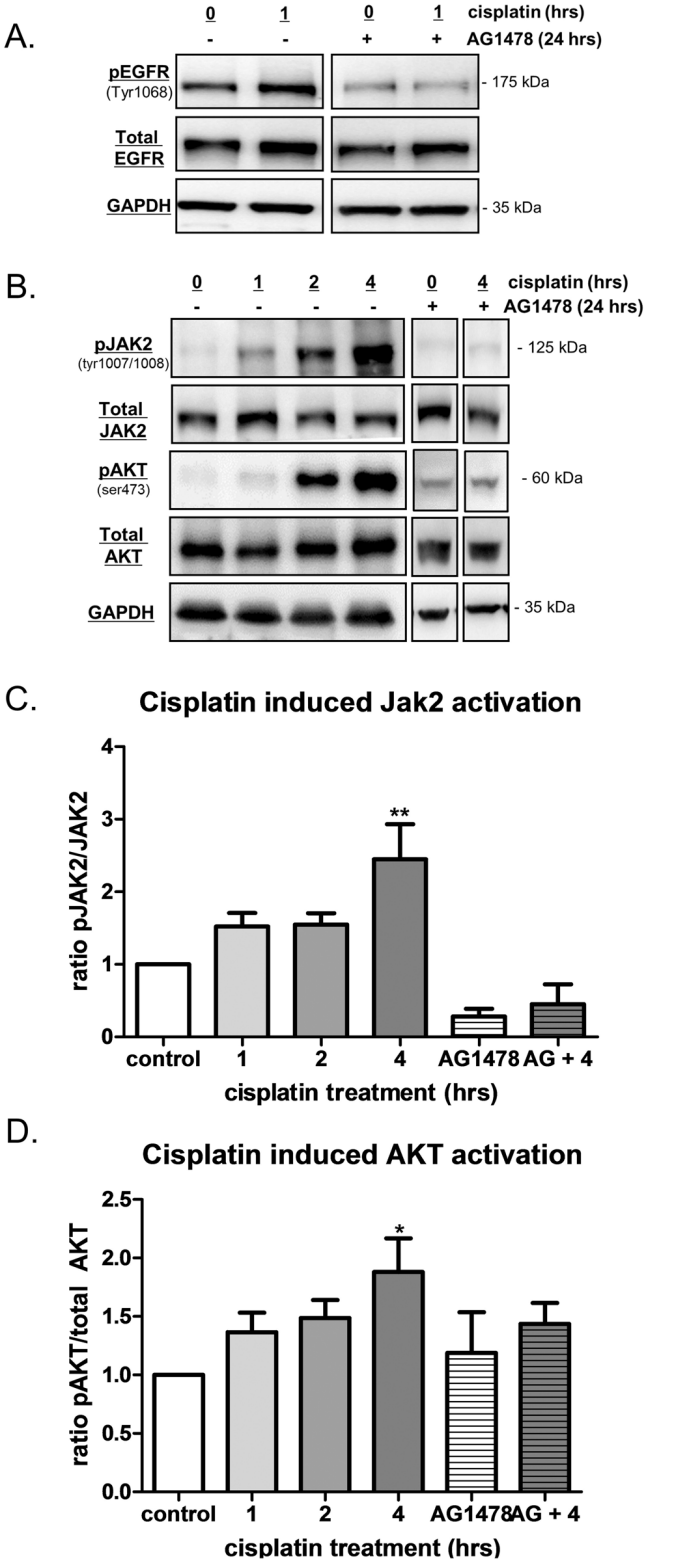
Acute cisplatin induces functional activation of the EGFR thereby activating downstream signaling pathways JAK2 and AKT. A) Representative western blots of pEGFR, Total EGFR and GAPDH of OVCA 433 cells-/+ 10 μM cisplatin for 1 hour in the presence and absence of the EGFR specific inhibitor AG1478 (2 μM, for 24 hour pre-incubation). B) Representative western blots for pJAK2 (tyr1007/1008), total JAK2, pAKT (ser473), total AKT and GAPDH. OVCA 433 cells-/+ 10 μM cisplatin for 1–4 hours and in presence of AG1478. The data generated for the following graphs represent at least 4 independent experiments i.e. ~4 different treatment/collection groups and ~4 different immunoblots. C) Ratio of JAK2 activation (pJAK2/total JAK2) data obtained after 10 μM cisplatin treatment for 1–4 hours and in the presence of AG1478, n = 4–6. Significant increases from control samples observed at 4 hours of cisplatin treatment. AG1478 pre-incubation for 24 hours blunted basal levels of activated JAK2 as well as cisplatin induced increases in JAK2 activation at 4 hours (AG+4). D) Ratio of AKT activation (pAKT/total AKT) data obtained after 10 μM cisplatin treatment for 1–4 hours and in the presence of AG1478, n = 4–7. Significant increases from control samples observed at 4 hours of cisplatin treatment. AG1478 pre-incubation for 24 hours blunted cisplatin induced increases in AKT activation at 4 hours (AG+4). One-way ANOVA revealed a significant effect of drug treatment and following Dunnet’s post hoc test significant differences from controls are indicated by *p<0.05, **p<0.01.

Several *in vitro* studies to date have used high doses of cisplatin (50–100 μM) to demonstrate increased activation of the EGFR over short treatment times [16–19]. However, studies looking at the pharmacokinetics in patients receiving 75–120 mg/m^2^ of cisplatin showed peak plasma levels between 0.2 to 14 μM [31,32], thereby suggesting that *in vitro* studies of 50–100 μM may not be clinically relevant. Here, we verified that cisplatin treatment at a more physiologically relevant dose (10 μM) activates the EGFR. Significant increases in EGFR phosphorylation (pEGFR) were observed in ovarian cancer cells (OVCA 433) following 30 min (0.5 h), 1 h and 2 h treatment with 10 μM cisplatin without changes to total EGFR (Fig 1A and 1B). The data were also expressed as a ratio of activated EGFR to total EGFR (Fig 1C) and found to be significantly increased at 2 h after cisplatin treatment. Specific inhibition of EGFR tyrosine kinase activity was achieved using AG1478 to determine the role of EGFR in cisplatin induced activation of downstream signaling pathways. As expected, AG1478 inhibited both basal and cisplatin induced levels of pEFGR without significant changes in total EGFR expression (Fig 2A). Functional activation of EGFR after cisplatin treatment was assessed by evaluation of downstream receptor targets following treatment with cisplatin and AG1478 (Fig 2B). JAK2 and AKT activation were increased significantly at the 4 h treatment time point when compared to untreated controls. Significant increases in ERK1/2 or STAT3 activation were not observed under these conditions (data not shown). Conversely, activation of JAK2 and AKT was greatly diminished by AG1478 while total protein levels remained relatively unchanged regardless of treatment (Fig 2B–2D). Thus, acute, physiologically relevant doses of cisplatin initiates EGFR signaling and activation of JAK2 and AKT.

### *In vitro* acquisition of cisplatin resistance

**Fig 3:**
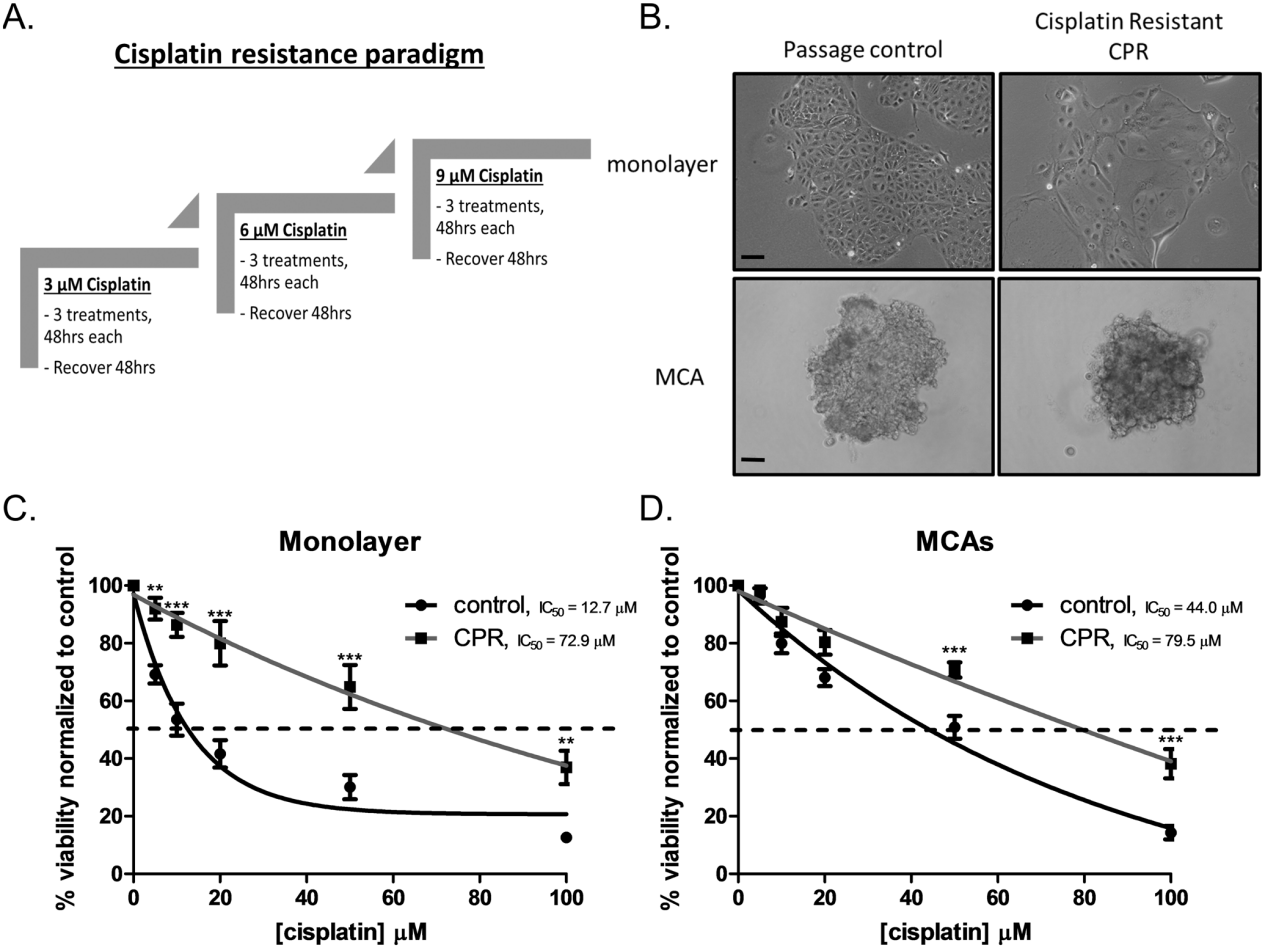
*In vitro* model of ovarian cancer platinum resistance. A) Schematic of cisplatin resistance paradigm as described in materials and methods. B) Representative 10X images of OVCA 433 control and cisplatin resistant (CPR) cells as a monolayer and as multicellular aggregates (MCA). Scale bar = 100 μm. C) Cell viability of monolayer control (black line), IC_50_ = 12.7 μM, and CPR (gray line) IC_50_ = 72.9 μM, in response to cisplatin treatment with doses [5, 10, 20, 50, 100 μM] for 48hrs. Data expressed as a percentage of untreated control cells, n = 8. Two-way ANOVA showed a significant overall main effect of cell type (control vs CPR) [F(1,84) = 88.10, p<0.001], a significant effect of cisplatin exposure [F(5,84) = 61.87, p<0.001] as well as a significant interaction [F(5,84) = 4.353, p<0.01]. Significant differences in viability observed at 5, 10, 20, 50, 100 μM cisplatin in control cells, **p<0.01, ***p<0.001. D) Cell viability of MCAs control (black line), IC_50_ = 44.0 μM, and CPR (gray line), IC_50_ = 79.5 μM, in response to cisplatin treatment with doses [5, 10, 20, 50, 100 μM] for 48hrs. Data expressed as a percentage of untreated control cells, n = 7. Two-way ANOVA showed a significant overall main effect of cell type (control vs CPR) [F(1,72) = 30.41, p<0.001], a significant effect of cisplatin exposure [F(5,72) = 128.3, p<0.001] as well as a significant interaction [F(5,72) = 3.946, p<0.01]. Significant differences in viability observed at 50, 100 μM cisplatin in control cells, ***p<0.001.

To evaluate the role of EGFR activation in the development of platinum resistance, we designed an *in vitro* paradigm of platinum resistance based on previously published work [20] (Fig 3A). Cisplatin resistant (CPR) cells grown under adherent (monolayer) conditions exhibited morphologic changes compared to control cells (Fig 3B). The literature suggests that 3D models, such as multicellular aggregates (MCAs), more accurately reflect ovarian cancer cell *in vivo* responses [33] and may be important in understanding drug resistance [34]. CPR MCAs were visibly more compact when compared to passage control MCAs (Fig 3B). This is consistent with our previous findings showing that compact MCAs are more resistant to cisplatin [22]. Monolayer cisplatin viability assays displayed a >5 fold increase in IC_50_ (control IC_50_ = 12.7 μM vs. CPR IC_50_ = 72.9 μM) (Fig 3C). Two-way ANOVA revealed a significant effect of cisplatin, significant effect of cell type (control vs. CPR) and a significant interaction. Viability assays for MCAs showed that CPR MCAs had increased in IC_50_ when compared to passage control MCAs (control MCA IC_50_ = 44.0 μM vs. CPR MCA IC_50_ = 79.5 μM) (Fig 3D). Again, two-way ANOVA revealed a significant effect of cisplatin, cell type and interaction. Thus, our *in vitro* model of platinum resistance proved to be a valuable tool in examining the molecular changes involved in the development of cisplatin resistance.

### Characterization of signaling pathways in CPR cells

**Fig 4:**
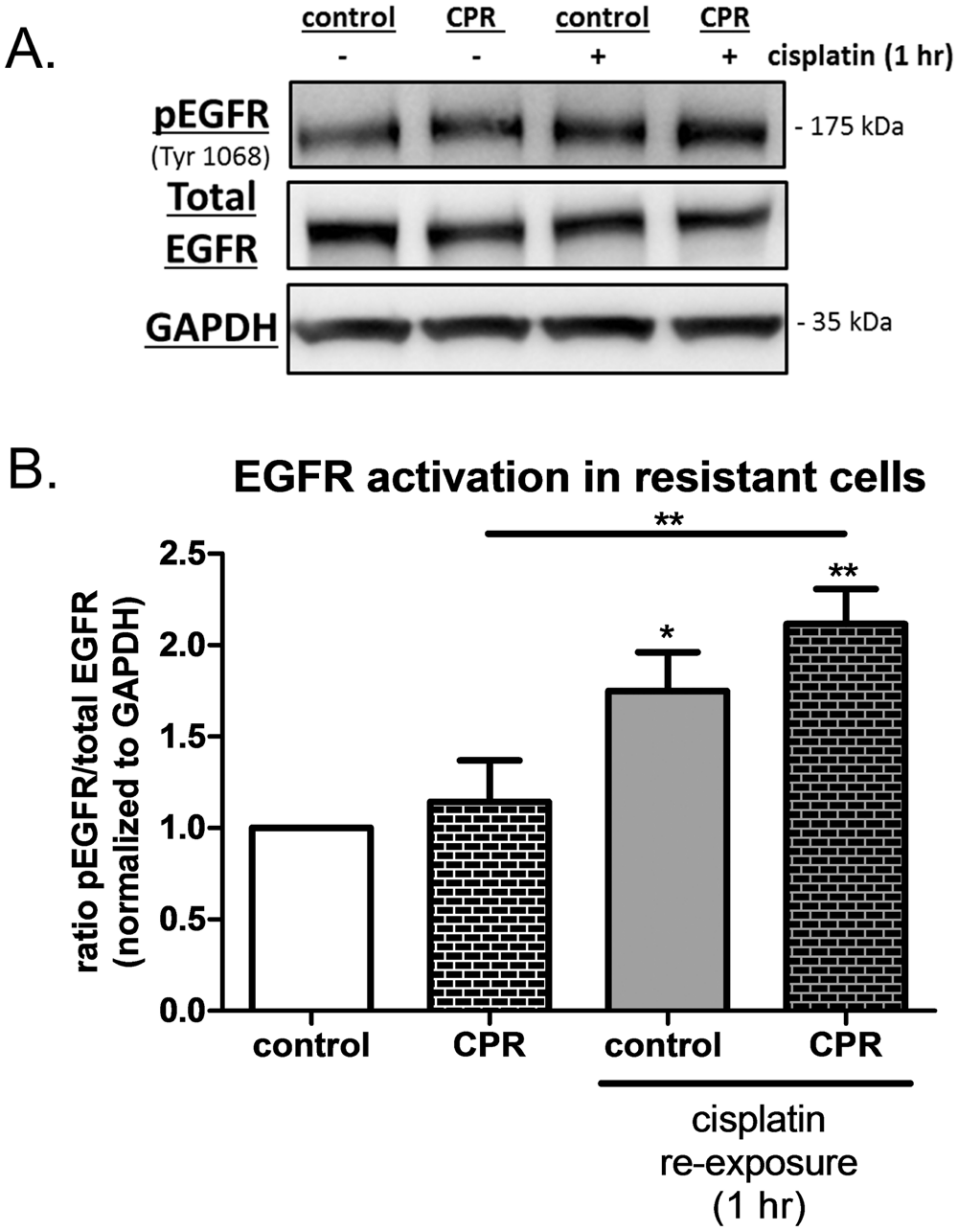
Cisplatin re-exposure induced EGFR activation in CPR cells. A) Representative western blots of pEGFR, Total EGFR and GAPDH for control and CPR cells-/+ 10 μM cisplatin (re-exposure) for 1 hour. B) Graphed data for the ratio of EGFR activation (pEGFR/total EGFR) in control and CPR cells after cisplatin re-exposure. One-way ANOVA followed by Tukey’s post hoc revealed significant increases in EGFR activation in control cells treated with cisplatin and in CPR cells treated with cisplatin compared to their untreated counterparts, but CPR cells were not significantly different than control cells in the absence of cisplatin. *p<0.05, **p,0.01.

The CPR cells remained resistant to further cisplatin treatment even in the absence of additional exposure to cisplatin demonstrating a persistent alteration in the CPR cells compared to their passage controls. While others reported that hyperactivity of the EGFR is associated with platinum resistance [20], we observed no difference in basal levels of pEGFR in CPR cells compared to control cells (Fig 4A). In addition, we found that CPR cells did not display significant increases in basal levels of EGFR downstream signaling pathways such as JAK2, AKT, STAT3, ERK1/2 (S1 Fig). However, the CPR cells remained susceptible to the cisplatin induced activation of the EGFR upon re- exposure to the drug for 1 hour, without concurrent changes to total EGFR levels (Fig 4A). CPR cells showed a significant increase in EGFR activation (ratio of pEGFR/total EGFR) when the cells were re-exposed to cisplatin (Fig 4B). Consistent with our observations in Fig 1, we show a significant increase in EGFR activation when control cells were treated with cisplatin, but there were no significant differences observed between control and CPR cells in the absence of cisplatin.

### Acute cisplatin treatment increases DNMT activity and this effect is dependent on EGFR activation

**Fig 5:**
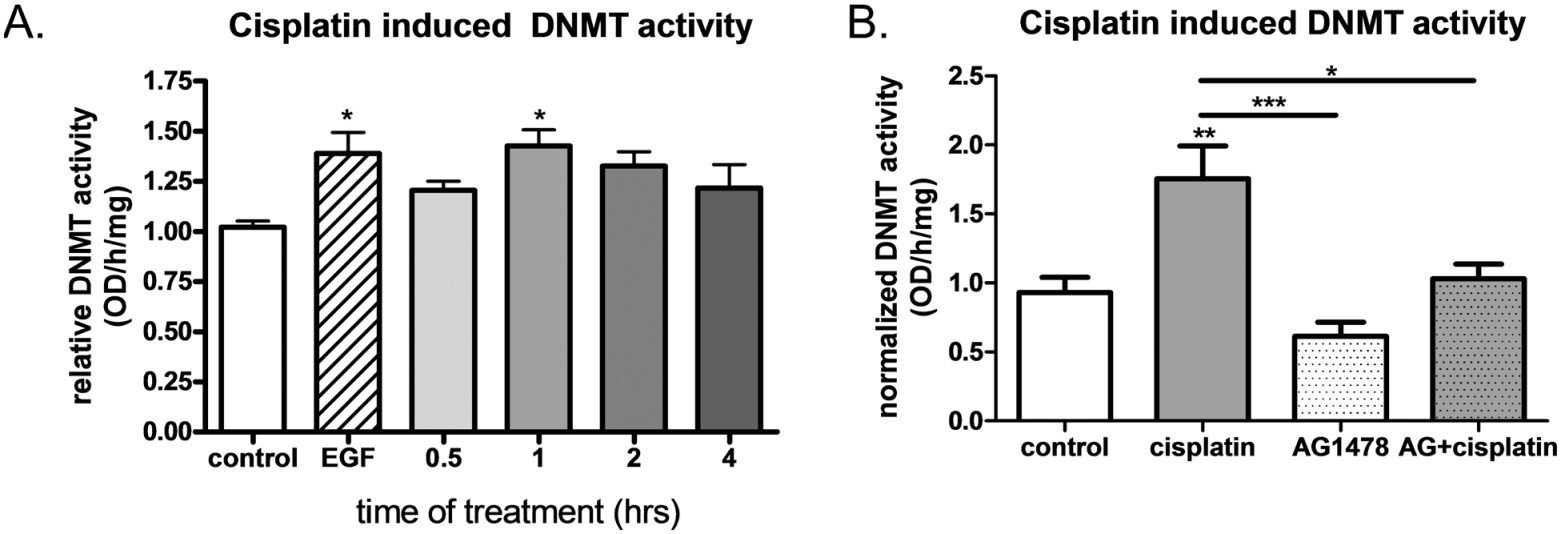
Acute cisplatin treatment increased DNMT activity and this effect was dependent on EGFR activation. A) Normalized DNMT activity following treatment with 10 μM cisplatin for 0–4 hours, n = 4. 10 nM EGF treatment for 15 minutes was used as a positive control. One-way ANOVA revealed a significant effect of cisplatin treatment and significant differences from control as determined by Dunnet’s post hoc analysis indicated by *p<0.05. B) Normalized DNMT activity following treatment with 10 μM cisplatin for 1 hour in the presence and absence of 2 μM AG1478 (24 hour pre-incubation), n = 5. One-way ANOVA followed by Tukey’s post hoc analysis revealed that the EGFR specific inhibitor AG1478 significantly attenuated cisplatin induced effects on DNMT activity. *p<0.05, **p<0.01, ***p<0.001.

Because EGFR activation increases DNMT activity and DNA methylation and there is an established link between DNA methylation and platinum resistance (27–32), we evaluated the effects of acute cisplatin exposure on DNMT activity. OVCA 433 cells treated with cisplatin showed significantly increased DNMT activity at 1 hour when compared to controls (Fig 5A). EGF treatment was used as a positive control in these studies as we previously showed increased DNMT activity under these conditions [11]. Additionally, inhibition of the EGFR with AG1478 prevented the cisplatin induced increase in DNMT activity at 1 hour (Fig 5B) indicating that this increase in DNMT activity is dependent on cisplatin’s activation of the EGFR. There were no significant differences observed between control samples and AG1478 alone treated samples, nor was there a significant difference between AG1478 alone and AG1478 +cisplatin groups.

### DNMT activity and global DNA methylation are increased in CPR cells, but EGFR inhibition diminishes this alteration

**Fig 6:**
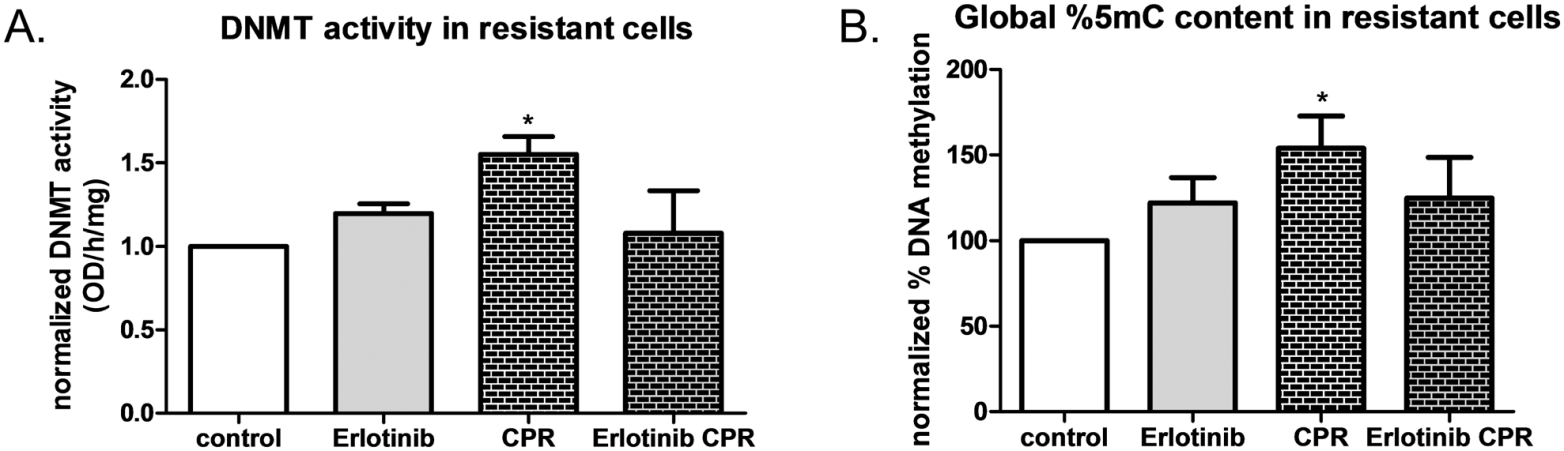
DNMT activity and global DNA methylation increased in CPR cells, but not in CPR cells under EGFR inhibition. A) Normalized DNMT activity in control, CPR cells as well as cells that received the EGFR inhibitor Erlotinib only or Erlotinib + cisplatin during the cisplatin resistance paradigm, n = 4. One-way ANOVA followed by Dunnet’s post hoc showed that Erlotinib co-treatment with cisplatin in the cisplatin resistance paradigm attenuated the increase in DNMT activity observed in CPR cells. B) Normalized percent 5-methylcytosine (5mC) or DNA global methylation for control, CPR cells as well as cells that received the EGFR inhibitor Erlotinib only or Erlotinib + cisplatin during the cisplatin resistance paradigm, n = 4–6. One way ANOVA followed by Tukey’s post hoc revealed a significant increase in global DNA methylation in CPR cells, but not in cells that received Erlotinib *p<0.05.

CPR cells showed a significant increase in DNMT activity compared to passage control cells (Fig 6A). So, we utilized the EGFR inhibitor, Erlotinib, to investigate whether DNMT activity could be prevented during cisplatin treatment. Erlotinib treatments alone did not affect DNMT activity, however, erotinib co-treatment with cisplatin during the platinum resistance paradigm (Erlotinib CPR) attenuated the cisplatin induced increase in DNMT activity. To evaluate the consequences of increased DNMT activity, we then measured global DNA methylation (total 5-methyl-cytosine content) in the experimental groups (Fig 6B). Data were normalized to values obtained for control cells and graphed as a percentage of the control. Total 5-methyl-cytosine content was significantly increased in CPR cells, but this increase was attenuated in cells under EGFR inhibition during the platinum resistance paradigm. These studies confirm that cisplatin induced alterations to DNMT activity and DNA methylation are dependent on EGFR activation. This increase in DNMT activity and DNA methylation in CPR cells could not be explained by increased levels of DNMTs present in those cells. In fact, analysis of DNMT levels in in control and CPR cells showed that DNMT1 and a phosphorylated form of DNMT1 at serine 714 (pDNMT1) previously linked to EGFR activation [35] are both significantly decreased in CPR cells (S2 Fig). DNMT3A, DNMT3B and DNMT3L all showed no significant differences in CPR cells compared to controls.

### EGFR inhibition reduces the extent of resistance observed in the in vitro model of platinum resistance

**Fig 7:**
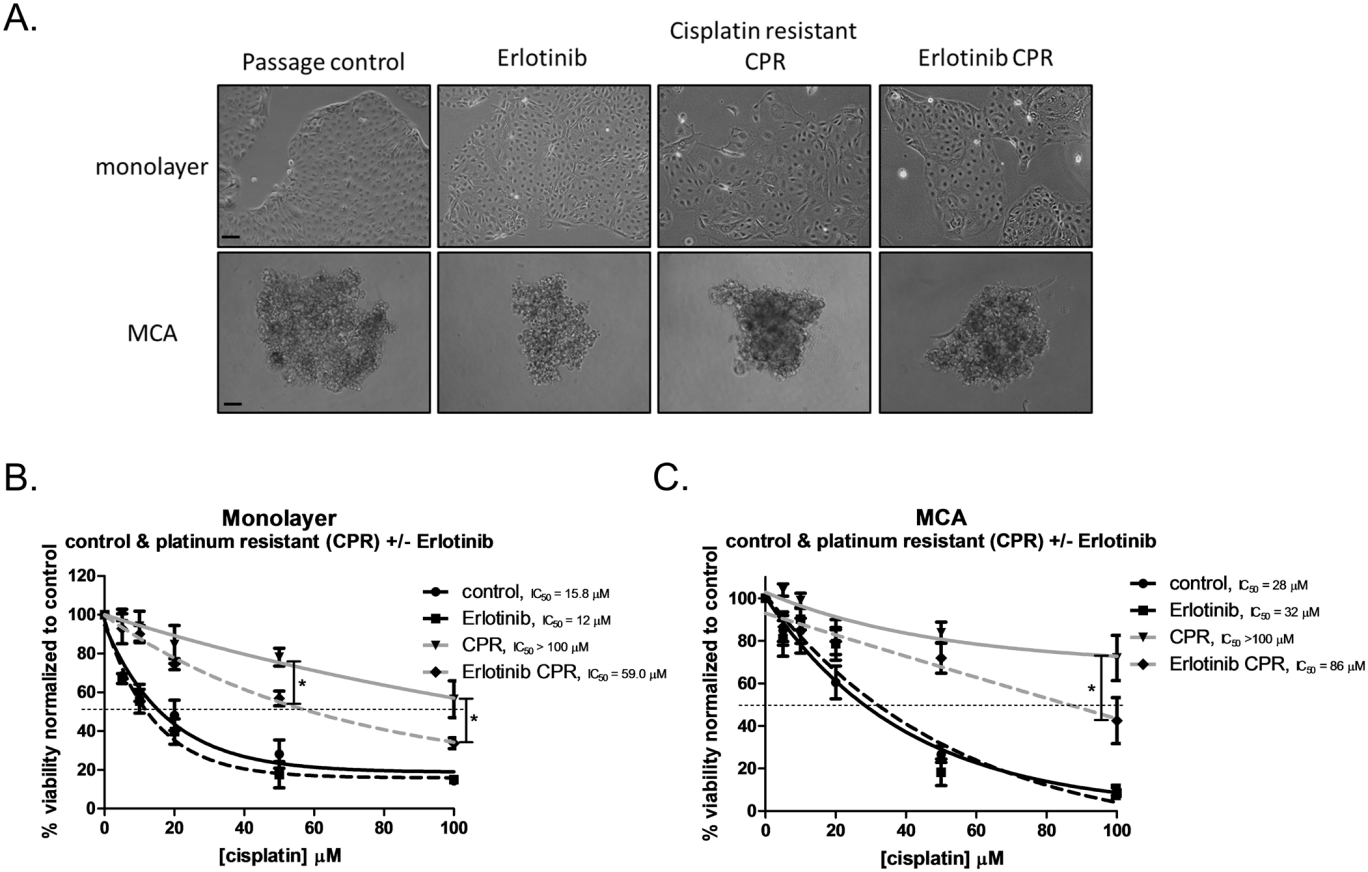
EGFR inhibition by Erlotinib attenuates the development of resistance conferred by the cisplatin resistance paradigm. A) Representative 10X images of OVCA 433 control, Erlotinib treated, cisplatin resistant (CPR) and cells that were given Erlotinib and cisplatin in the platinum resistant paradigm (Erlotinib CPR) as a monolayer and as multicellular aggregates (MCA). Scale bar = 100 μm. B) Cell viability of monolayer control (black line), IC_50_ = 15.8 μM, CPR (gray line) IC_50_ >100 μM, Erlotinib alone (dotted black line), IC_50_ = 12 μM, and Erlotinib CPR (dotted gray line) IC_50_ = 59 μM, in response to cisplatin treatment with doses [5, 10, 20, 50, 100 μM] for 48hrs. Data expressed as a percentage of untreated control cells, n = 4. Two-way ANOVA showed a significant overall main effect of cell type (control vs. Erlotinib vs. CPR vs Erlotinib CPR) [F(3,72) = 65.88, p<0.001], a significant effect of cisplatin exposure [F(5,72) = 94.46, p<0.001] as well as a significant interaction [F(15,72) = 3.468, p<0.001]. Significant differences between CPR and Erlotinib CPR were observed at 50 & 100 μM cisplatin, *p<0.05. C) Cell viability of MCA control (black line), IC_50_ = 28 μM, CPR (gray line) IC_50_ >100 μM, Erlotinib alone (dotted black line), IC_50_ = 32 μM, and Erlotinib CPR (dotted gray line) IC_50_ = 86 μM, in response to cisplatin treatment with doses [5, 10, 20, 50, 100 μM] for 48hrs. Data expressed as a percentage of untreated control cells, n = 4–5. Two-way ANOVA showed a significant overall main effect of cell type (control vs. Erlotinib vs. CPR vs Erlotinib CPR) [F(3,84) = 29.37, p<0.001], a significant effect of cisplatin exposure [F(5,84) = 70.02, p<0.001] as well as a significant interaction [F(15,84) = 5.609, p<0.001]. A significant difference between CPR and Erlotinib CPR were observed at 100 μM cisplatin, *p<0.05.

Given the evidence that using DNMT inhibitors reverses platinum resistance [6–10] as well as our observations that EGFR inhibition attenuated DNMT activity and DNA methylation in CPR cells, we tested whether this resulted in a change in platinum sensitivity. As initially demonstrated in Fig 3, we found in a separate set of experiments that CPR cells are significantly more resistant than their passage control counterparts grown as monolayer (control IC_50_ = 15.8 μM vs. CPR IC_50_ greater than 100 μM) (Fig 7B) and MCAs (control IC_50_ = 28 μM vs. CPR IC_50_ greater than 100 μM) (Fig 7C). Inhibition by Erlotinib alone does not affect sensitivity to cisplatin; monolayer (Erlotinib IC_50_ = 12 μM) and MCAs (Erlotinib IC_50_ = 32 μM). EGFR inhibition with Erlotinib during the platinum resistance paradigm reduces the degree of resistance observed in CPR cells for both monolayer (CPR IC_50_ greater than 100 μM vs. Erlotinib CPR IC_50_ = 59 μM) and MCAs (CPR IC_50_ greater than 100 μM vs. Erlotinib CPR IC_50_ = 86 μM). Two-way ANOVA revealed a significant effect of cell type (control vs. Erlotinib vs. CPR vs Erlotinib CPR), a significant effect of cisplatin treatment and a significant interaction. Post hoc analysis revealed significant differences between CPR and Erlotinib at 50 μM and 100 μM. Taken together, this suggests that the EGFR plays a role in the development of platinum resistance as EGFR inhibition diminished the amount of resistance that was achieved by our model of platinum resistance.

## Discussion

Platinum resistance is a significant problem in ovarian cancer. Alterations in DNA methylation are commonly seen in association with resistance to cisplatin [24–28] and DNA methylation has been proposed as a mechanism underlying the development of platinum resistance [27,36]. Because DNA hypermethylation (increased methylation) is a more frequent occurrence than hypomethylation (or loss of methylation) during the acquisition of platinum resistance [27], the use of demethylating agents to combat platinum resistance is an emerging approach. *In vitro* studies with ovarian cancer cell lines have shown that treatment with demethylating agents successfully demethylate resistant cells and resensitize those cells to cisplatin [10,37,38]. Demethylating agents also reactivate tumor suppressor genes, RASSF1A, and putative drivers of chemoresistance, MLH1 and ZIC1 in cells [37]. Clinical trials with heavily pretreated and platinum-resistant ovarian cancer patients revealed that low dose treatment with a demethylating agent, decitabine, combined with carboplatin is tolerated well [8]. Treatment results in DNA demethylation in peripheral blood and tumors, and induced 35% objective response rate and progression free survival (PFS) of 10.2 months with 53% of patients free of progression at 6 months [6–8]. A PFS of 10.2 months with decitabine indicates a significant improvement compared to another clinical trial of patients with resistant disease which showed that chemotherapy alone resulted in a median PFS of 3.4 months [39]. Another phase II trial with decitabine and carboplatin showed no benefit for patients [36], but it did suggest that prolonged treatment with low dose demethylating agents as seen in other studies [7] may be required to achieve favorable clinical responses. Taken together, these preclinical and clinical studies suggest that DNA methylation is a key mechanism underlying platinum resistance. The goal of this study was to identify and describe a mechanism and potential target to prevent, or attenuate, the development of resistance during platinum drug treatment.

Experimental and clinical observations demonstrating the development of resistance following repeated cycles of platinum treatment highlights the need to evaluate mechanisms by which cisplatin contributes to resistance. The results presented here depict the EGFR as a mediator of platinum induced alterations leading to the development of platinum resistance. We show that cisplatin treatment at clinically relevant doses functionally activates the EGFR and its downstream signaling pathways, JAK2 and AKT; both of which have been previously implicated in platinum resistance [20,40]. Through activation of the EGFR, cisplatin also increased DNMT activity. Cells undergoing repeated cisplatin treatment developed resistance to the drug, showed increased DNMT activity and increased global DNA methylation. However, inhibition of the EGFR during repeated cisplatin treatment attenuated drug resistance and prevented increases in DNMT activity and global methylation. This is consistent with our previous findings showing the EGFR as a regulator of DNA methyltransferase activity and DNA methylation [11] and substantiates the EGFR as a novel regulator of DNA methylation changes associated with the development of platinum resistance. These data suggest that EGFR inhibition during cisplatin treatment reduces the level of resistance achieved by preventing alterations to DNA methylation, as opposed to reversing the alterations to DNA methylation after the fact. Identification of tangible targets, such as the EGFR, responsible for the development of platinum resistance could provide a means to make platinum therapy more effective for ovarian cancer and other cancers.

In addition, AKT has previously been shown to phosphorylate DNMT1 and promote its stability thereby potentially having an effect on DNMT activity [41]. Inhibition of AKT activation or downregulation of AKT resensitizes resistant ovarian cancer cells to cisplatin [40,42]. While we found significant increases in JAK2 activation with cisplatin treatment, we found no significant alterations to ERK1/2 or STAT3 in our studies. Interestingly, inhibition of JAK/STAT or ERK1/2 have been shown to downregulate expression of DNMT3L [43] or DNMT1 [44], respectively; hence conceivably contributing to regulation of DNMT activity and DNA methylation. Since multiple signaling molecules likely regulate this process, upstream inhibition at the level of the EGFR confers inhibition of downstream signaling and our studies are consistent with this concept; thus the focus remained at the level of the EGFR. Ultimately, a better understanding of mechanisms driving the acquisition of platinum resistance will enable us to provide better overall treatment to cancer patients. We acknowledge that cisplatin within the cell is likely affecting additional intracellular signaling pathways as cisplatin is known to increase free radical generation [45] which has been shown to regulate receptor tyrosine kinase [46] and EGFR activation [47]. Furthermore, the EGFR is a member of the ErbB family of RTKs therefore is one part of a much larger signaling network and can have crosstalk with other pathways and RTKs [48]. However, the primary focus of this paper was to characterize the contribution of the EGFR in the development of acquired platinum resistance; thus, the involvement of other RTKs is an area of current investigation.

In our studies, Erlotinib treatment during the platinum resistance paradigm attenuated the resistance observed in both monolayer and MCA cultures, indicating that EGFR inhibition is a relevant target in the acquisition of platinum resistance. We used two different EGFR specific tyrosine kinase inhibitors (TKIs) AG1478 and Erlotinib. Both AG1478 and Erotinib are quinazolines [49], inhibit the EGFR by binding the ATP site thereby preventing autophosphorylation of the receptor [50] and have been linked to reversal of drug resistance [49,51] or modulation of drug resistance in ovarian cancer [50,52]. Erlotinib has been approved for treatment in non-small cell lung cancer (NSCLC) [4,53] and has been used in several clinical trials for ovarian cancer [4,50,52–54]. Erlotinib, either as a single agent in first line treatment or as maintenance therapy, in ovarian cancer patients did not show significant benefits nor was Erlotinib able to reverse resistance in platinum resistant patients when combined with platinum-based therapy (see [50] for review). Of the few studies evaluating the effects of Erlotinib in combination with platinum-taxol as first-line therapy Blank *et al*. found no improvements to pathologic complete response; however, median PFS for patients who received Erlotinib/carboplatin/paclitaxel first-line treatment was 34.3 months [54]. In comparison, another study evaluating Erlotinib treatment after first line chemotherapy showed a median PFS of only 12.7 months for patients receiving platinum based first line therapy followed by Erlotinib maintenance therapy [55]. This supports a potential benefit to EGFR inhibition during first-line treatment (prior to the development of platinum resistance) in prevention of acquired resistance.

In conclusion, we show that the EGFR pathway plays an important role in the development of platinum resistance. We highlight this pathway as a novel regulator of DNA methylation associated with the development of platinum resistance and we demonstrate that inhibition of this pathway attenuates resistance observed in a model of platinum resistance. As opposed to studies focused on reversing platinum resistance with demethylating agents, we provide evidence suggesting that we can target pathways to prevent the development of resistance. This work provides a targetable, mechanistic link between cisplatin treatment and platinum resistance that may repurpose EGFR inhibitors in ovarian cancer.

## Supporting Information

S1 Fig CPR cells do not innately show increased activity in signaling pathways downstream of the EGFR. A) Representative western blots of control and CPR cells for pJAK2, total JAK2, pSTAT3, total STAT3, pAKT, total AKT, pERK1/2, total ERK1/2 and GAPDH. B) JAK2 activation (Ratio of pJAK2/total JAK2), n = 10. C) STAT3 activation (Ratio STAT3/total STAT3), n = 8. D) AKT activation (Ratio pAKT/total AKT), n = 9. E) ERK1/2 activation (Ratio pERK1/2 / total ERK1/2), n = 8.
(TIF)

S2 Fig CPR cells do not display alterations in DNMT protein levels that would explain associated changes in DNMT activity. A) Representative western blots of control and CPR cells for DNMT1, pDNMT1 DNMT 3A, DNMT3B, DNMT 3L and GAPDH. B) DNMT1 significantly decreased in CPR cells, n = 9, ***p<0.001.C) pDNMT1 (ser714) significantly decreased in CPR cells, n = 5, *p<0.05. D) DNMT3A, n = 9. E) DNMT3B, n = 5. F) DNMT3L, n = 5.
(TIF)
