# Supplementary figures and images for: Contributions of the Epidermal Growth Factor Receptor to Acquisition of Platinum Resistance in Ovarian Cancer Cells
Source: PLoS One. 2015 Sep 9;10(9):e0136893. doi: 10.1371/journal.pone.0136893 (PMC4564275; doi:10.1371/journal.pone.0136893)

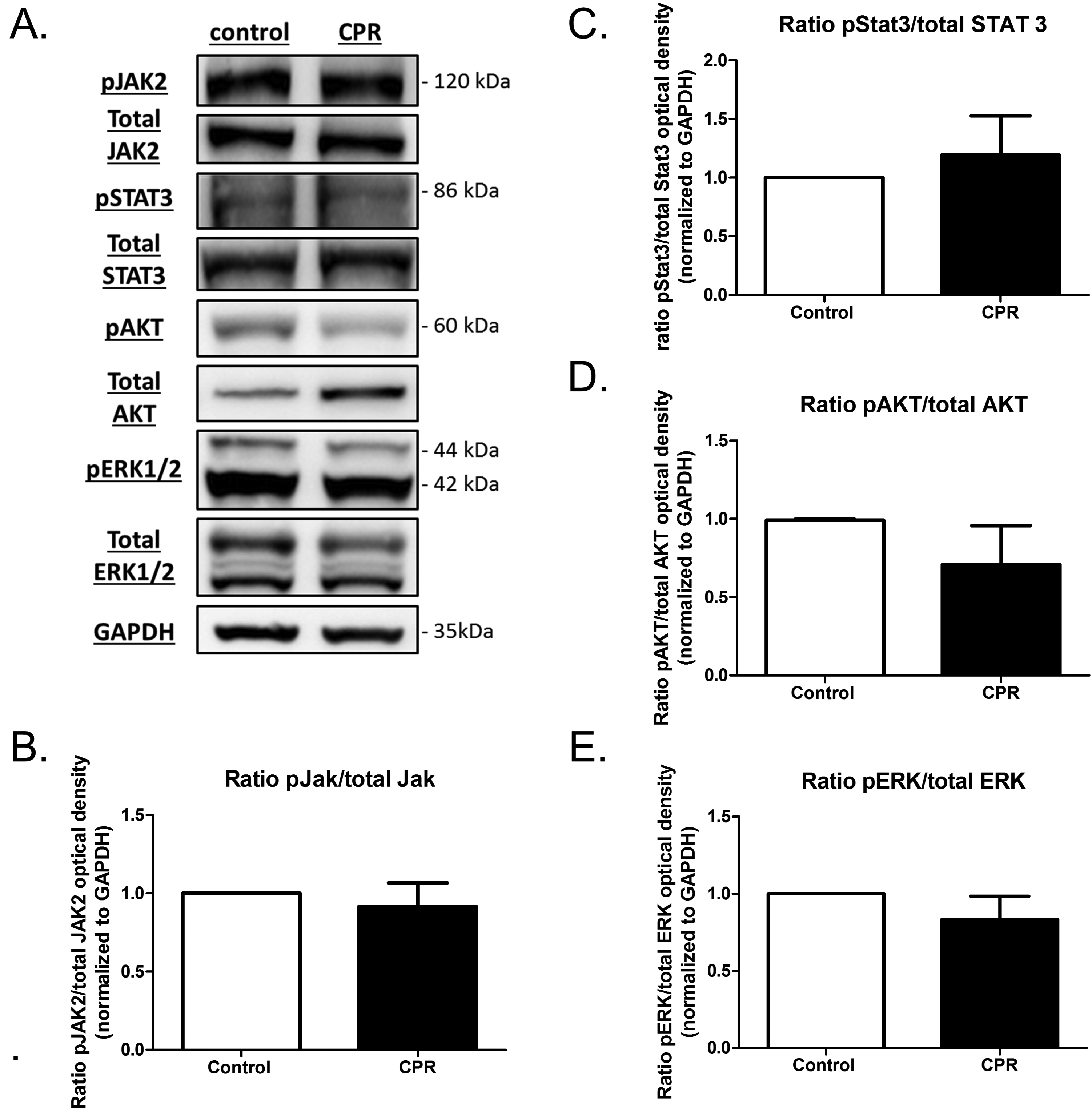

Supplement: S1 Fig — A) Representative western blots of control and CPR cells for pJAK2, total JAK2, pSTAT3, total STAT3, pAKT, total AKT, pERK1/2, total ERK1/2 and GAPDH. B) JAK2 activation (Ratio of pJAK2/total JAK2), n = 10. C) STAT3 activation (Ratio STAT3/total STAT3), n = 8. D) AKT activation (Ratio pAKT/total AKT), n = 9. E) ERK1/2 activation (Ratio pERK1/2 / total ERK1/2), n = 8. (TIF) [file pone.0136893.s001.tif]

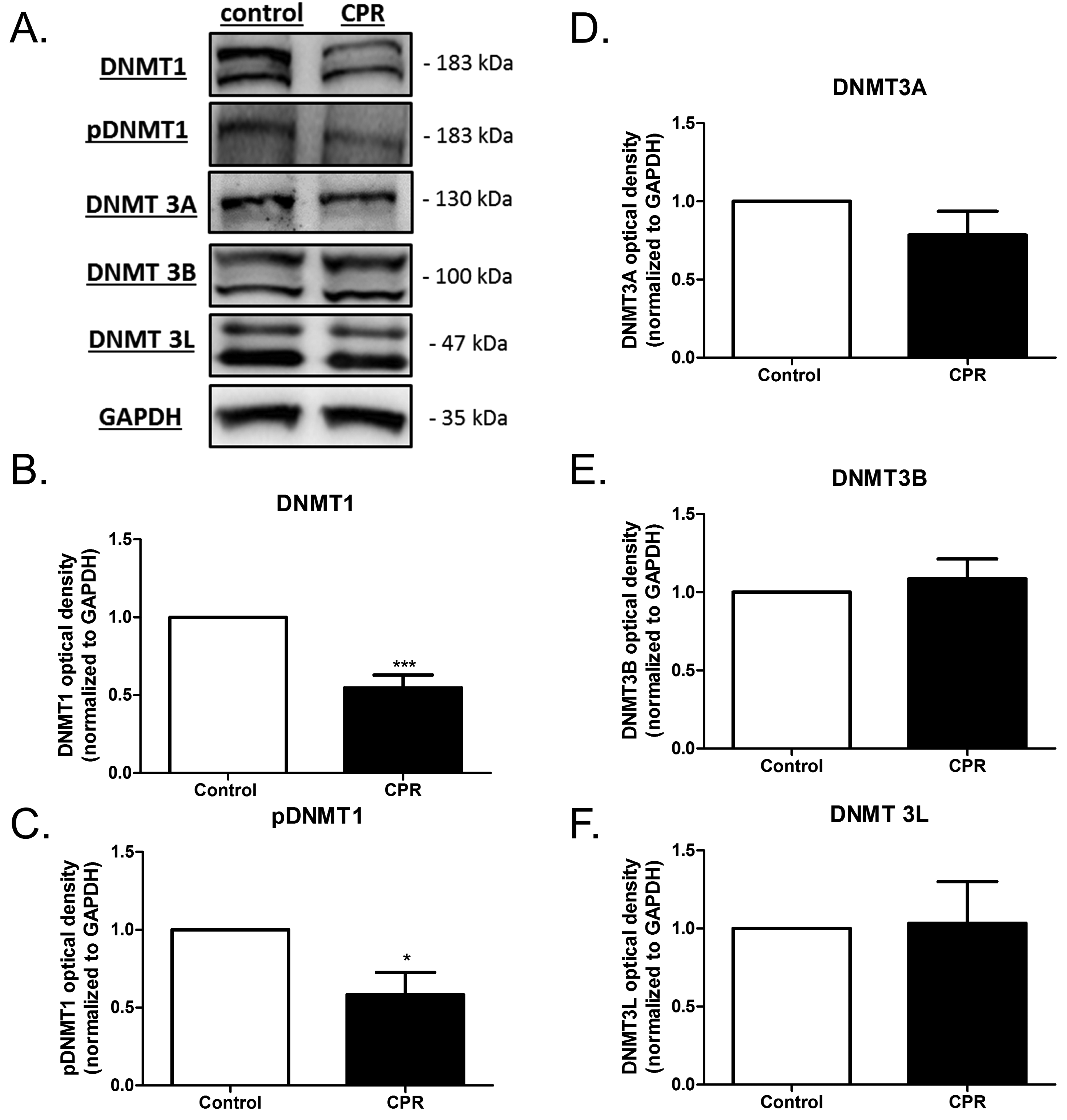

Supplement: S2 Fig — A) Representative western blots of control and CPR cells for DNMT1, pDNMT1 DNMT 3A, DNMT3B, DNMT 3L and GAPDH. B) DNMT1 significantly decreased in CPR cells, n = 9, ***p<0.001.C) pDNMT1 (ser714) significantly decreased in CPR cells, n = 5, *p<0.05. D) DNMT3A, n = 9. E) DNMT3B, n = 5. F) DNMT3L, n = 5. (TIF) [file pone.0136893.s002.tif]
